# An Evaluation of the Relationship between Training of Health Practitioners in a Person-Centred Care Model and their Person-Centred Attitudes

**DOI:** 10.5334/ijic.7564

**Published:** 2023-11-24

**Authors:** Esther Li Ping Lim, Rebecca Hui Shan Ong, Johan Thor, Monika Allgurin, Boel Andersson Gäre, Julian Thumboo

**Affiliations:** 1 School of Health and Welfare, Jönköping University, Jönköping, Sweden; 2 Allied Health Division, Singapore General Hospital, Singapore; 3 Centre for Person-centred Care, Singapore Health Services, Singapore; 4 Population Health and Integrated Care Office, Singapore General Hospital, Singapore; 5 Health Services Research, Changi General Hospital, Singapore; 6 Jönköping Academy for Improvement of Health and Welfare, Jönköping University, Jönköping, Sweden; 7 Futurum, Region Jönköping County, Jönköping, Sweden; 8 SingHealth Office of Regional Health, Singapore Health Services, Singapore; 9 SingHealth Centre for Population Health Research and Implementation, Singapore Health Services, Singapore; 10 Department of Rheumatology and Immunology, Singapore General Hospital, Singapore

**Keywords:** person-centred care, integrated care, power, advocacy, training, coproduction, practitioners

## Abstract

**Introduction:**

The Esther Network (EN) person-centred care (PCC) advocacy training aims to promote person-centred attitudes among health practitioners in Singapore. This study aimed to assess the relationship between the training and practitioners’ PCC attributes over a 3-month period, and to explore power sharing by examining the PCC dimensions of “caring about the service user as a whole person” and the “sharing of power, control and information”.

**Methods:**

A repeated-measure study design utilising the Patient-Practitioner Orientation Scale (PPOS), was administered to 437 training participants at three time points – before training (T1), immediately after (T2) and three months after training (T3). A five-statement questionnaire captured knowledge of person-centred care at T1 and T2. An Overall score, Caring and Sharing sub-scores were derived from the PPOS. Scores were ranked and divided into three groups (high, medium and low). Ordinal Generalised Estimating Equation (GEE) model analysed changes in PPOS scores over time.

**Results:**

A single, short-term training appeared to result in measurable improvements in person-centredness of health practitioners, with slight attenuation at T3. There was greater tendency to “care” than to “share power” with service users across all three time points, but the degree of improvement was larger for sharing after training. The change in overall person-centred scores varied by sex and profession (females score higher than males, allied health showed a smaller attenuation at T3).

**Conclusion:**

Training as a specific intervention, appeared to have potential to increase health practitioners’ person-centredness but the aspect of equalising power was harder to achieve within a hierarchical structure and clinician-centric culture. An ongoing network to build relationships, and a supportive system to facilitate individual and organisational reflexivity can reinforce learning.

## Introduction

Globally, new models of care are being adopted to provide high quality health and welfare services, especially in response to an ageing population. In this context, a ‘person-centred’ definition of integrated care that is coproduced with, and focused on what is important to, service users, provides a particularly compelling logic [1,2,3,4]. This approach requires practitioners to provide care by listening to what matters to, and share power with, service users [5] so that service users “can play an active role in producing services of consequence to them” [6 p1073].

Person-centred care (PCC) has been shown to improve clinical outcomes [7,8,9], especially for chronic conditions [7,10], and requires careful and intentional implementation in clinical practice. However, it is a challenge to have PCC consistently practiced by health practitioners because of the previous emphasis on disease-centric practice and the lack of systems to integrate PCC into clinical practice [11]. Nevertheless, the compelling evidence for the shift in paradigm from a disease-centric to PCC model has led to calls for the incorporation of PCC training into health practitioners’ learning curriculum [12,13]. PCC training in healthcare encourages shared control and care decision-making with service users through coproduction, and a focus on the patient as a whole person with values, needs and preferences situated in individual circumstances [14]. Of note, caring improves satisfaction but it is the sharing of power, control and information that improves adherence [7]. Within the scope of PCC, there have been many interventions aimed at improving healthcare communication, but few have truly embraced the practice of service users having more power, control and information in their own care [4].

### Esther Network (EN) PCC Advocacy Training

Originating from Sweden, the EN model promotes PCC in the form of equal partnership and sharing of power with service users through coproduction of care services, guided by the core question “What is best for Esther?” “Esther” is a figurative persona of a person with complex needs [15]. To create value, service users such as Esthers, need to be treated as active partners by health practitioners, to achieve care goals that matter most to them [4,16,17]. The concept of advocacy training was developed from the idea of practitioner-advocates acting as intermediaries for quality improvement to close the gap between policy and practice [18]. It describes how healthcare transformation can happen when, at all levels of the system, change-making becomes a part of everyone’s daily job [19]. The EN concept was adopted and adapted by Singapore Health Services (SingHealth) in 2016 [20]. Equipping of practitioners was identified in our previous study as one of the key mechanisms that enabled individuals to own a shared understanding of PCC. Thus, an adaptation made in EN Singapore was a two-hour focused training (the EN PCC advocacy training) for health practitioners, anchored by the central elements of patient involvement and coproduction, to raise awareness of PCC that health practitioners could practise in their daily work [20].

Previous research has shown differences in person-centred orientation related to practitioners’ characteristics such as sex, work experience, professional background [21,22,23]. Evidence suggests that attitudes towards person-centredness represent complex interplays of context, culture and socio-demographic variables [10]. Studying the person-centred orientation of Singapore medical students, Lee et al [10] found that in terms of showing care, they were comparable with their American counterparts, but were less inclined to share power, information and knowledge with patients or believed that patients do not expect them to.

To our knowledge, there is very limited data on the efficacy of PCC training in changing attitudes towards patient-centred care among health practitioners. Also, factors associated with changes in person-centred scores over time have not been widely examined. Understanding the effect of the training, and factors related to changes over time can be used to further develop advocacy training that may encourage attitude shifts towards PCC.

This study therefore aimed to evaluate the impact of a training session in PCC for health practitioners on participants’ person-centred attitudes over time. The person-centred attitudes included caring for, and sharing of power, control and information with, service users. The primary research question was: to what extent did PCC advocacy training coincide with changes in the person-centredness of healthcare practitioners? Secondly, we sought to explore the relationship between practitioners’ characteristics and person-centredness. Therefore, the primary aim of this study was to evaluate the relationship between training and PCC attitudes among health practitioners. In the process, we examined the influence of practitioners’ characteristics (sex, profession and work experience) and time on person-centredness.

## Methods

### Context

SingHealth is an integrated healthcare network of 13 institutions with four acute hospitals, three community hospitals, five national specialty centres and a primary care network with nine polyclinics, focused on providing healthcare services to the Eastern region of Singapore. Its workforce consists of 4343 doctors, 11,709 nurses, 6735 allied health professionals [24]. In 2016, SingHealth leaders supported the adoption of a PCC model known as the “Esther Network”. Arising from this, the PCC advocacy training was developed to address an unmet need in training all patient-fronting clinical staff in PCC. It was intentionally designed as a focused two-hour session to equip all health practitioners with an understanding of a PCC model in SingHealth.

**Figure 1:**
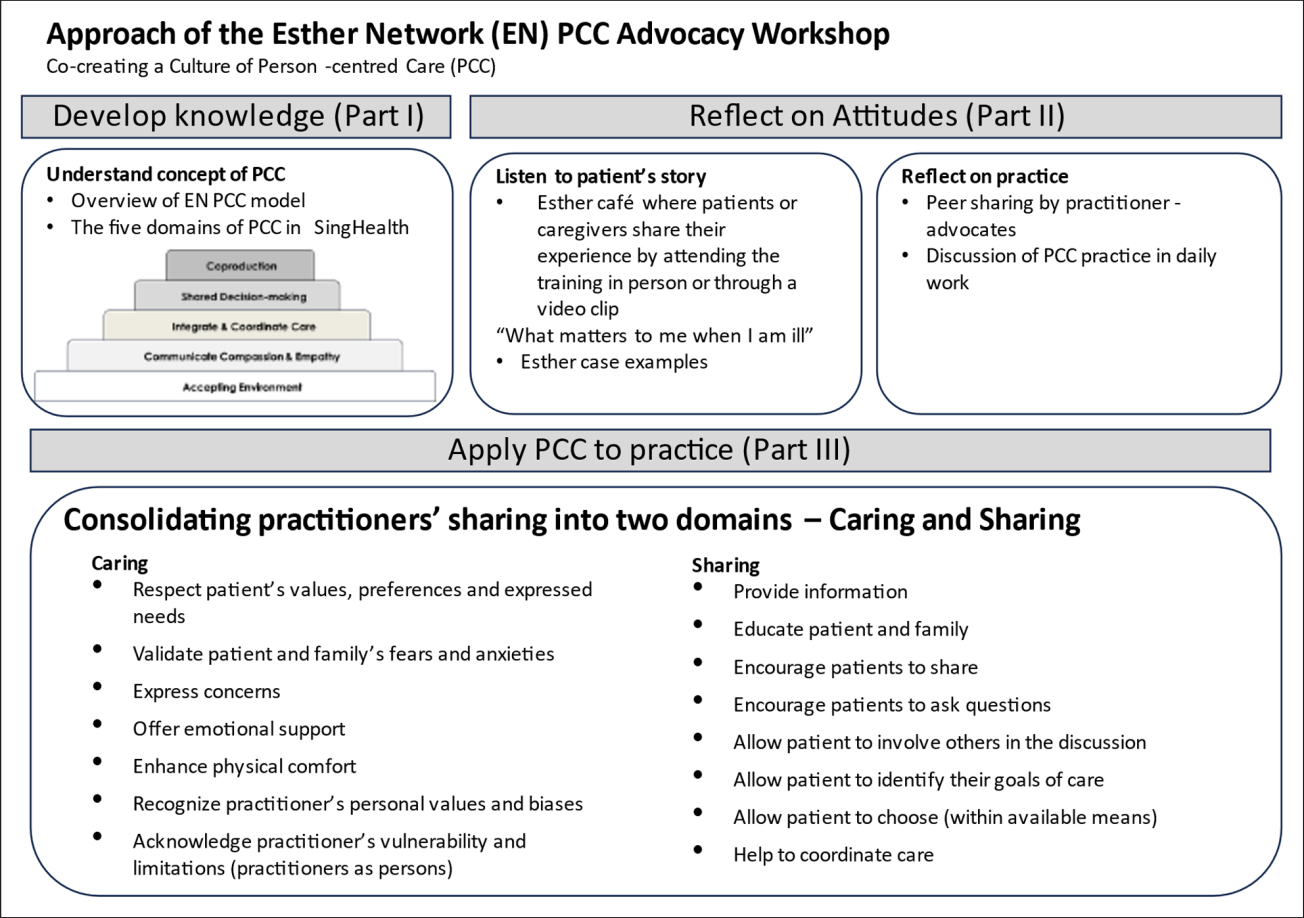
The Esther Network PCC Advocacy Training.

The purpose of the training was to train “practitioner-advocates” to advocate for PCC practice at the workplace. In total, 17 advocacy training sessions were conducted for doctors, nurses and allied health professionals during the period August 2020 to Nov 2021. There are three parts to the training (Figure 1). The highlights are: an “Esther Café” where service users shared their experience of healthcare and what mattered to them when ill; definition of PCC adopted by SingHealth that included both the “Caring” and “Sharing” components; facilitated discussions and reflection of practitioners’ experience in applying PCC.

### Study Design

**Figure 2:**
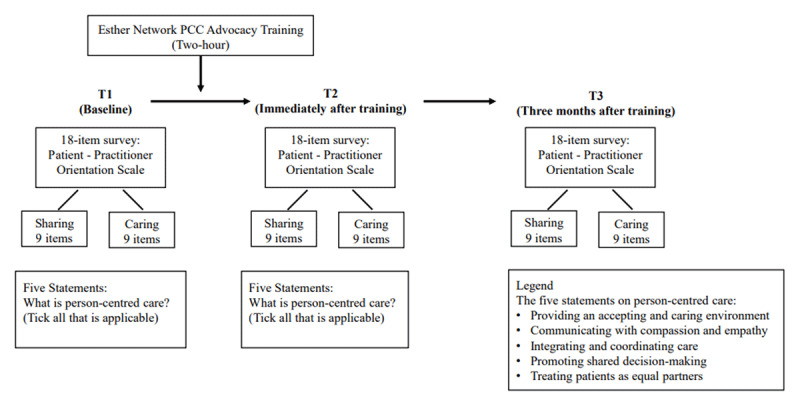
Research design. #PCC, person-centred care.

In a single arm pre-post design, Krupat’s 18-item Patient-Practitioner Orientation Scale (PPOS) questionnaire [25] was administered to attendees of the training who consist of SingHealth doctors, nurses and allied health professionals to document changes in person-centred attitudes and knowledge amongst them across three time points (T1 – baseline i.e. immediately before the training, T2 – immediately post training and T3 – three months after training) (Figure 2). Immediately before and immediately post training, participants also answered a self-administered five-statement questionnaire developed by the study team, to capture understanding of person-centred care using definitions of various aspects of PCC (Figure 2). The responses were collected anonymously (i.e. without any identifiers). Ethical approval was obtained from the SingHealth Institutional Review Board (Reference number: 2021/2064).

### Participants

All participants who attended the training between August 2020 and November 2021 were invited to participate in this study. Participants who agreed to the study provided written consent. Data was collected from August 2020 to February 2022. The eligibility criteria were (i) aged 21 years and above, (ii) completed the training session, and (iii) able to understand and answer the English questionnaire.

### Study Instruments

#### Patient-Practitioner Orientation Scale

The PPOS consists of 18-items in a six-point Likert (strongly agree-strongly disagree) format. An “Overall” score ranging from patient-centric to practitioner-centric can be calculated in addition to two sub-scores: the “Sharing” sub-scale carries nine items that reflect the extent to which respondents believe that a) practitioners and patients should share power and control on a relatively equal basis, and b) that practitioners should share as much information with their patients as possible. The second nine-item sub-scale “Caring” refers to the extent that respondents believe that a) caring about the emotions and good interpersonal relations is a key aspect of the medical encounter, and b) that practitioners should care about the patient as a whole person rather than as a medical condition. [26].

The PPOS has been validated in multiple studies worldwide [27] among doctors [10,26,28], nurses [29] and allied health professionals [22,30]. It has adequate psychometric properties and has demonstrated acceptable internal consistency; alpha ranging from 0.48 to 0.72 for sharing, 0.42 to 0.53 for caring, and 0.62 to 0.88 for overall score [26,31,32,33]. Construct validity of the PPOS scores has also been supported across studies among various health practitioners and practices, such as medicine and physical therapy [28,30,33]. The PPOS has also undergone extensive cross-cultural adaptations in Asia, Europe, and South America [10,21,32,34,35,36,37].

### Statistical Analysis

The primary outcome measure was the change in PPOS scores from baseline to 3^rd^ month. Assuming a 5% error margin, to have 80% statistical power to detect a Cohen’s D effect size of 0.2 at 3^rd^ month, 199 participants would be required. Assuming a 20% drop-out rate, we needed a sample of at least 250 participants for this study. Sample size calculations were performed with G*Power (ver. 3.1.9.4) [38].

Data was analysed using R for Windows, version 4.0.5. Continuous data were presented as means and standard deviations (SD), and categorical data were presented as frequencies and percentages. The student’s t-test, chi-square, or Fisher’s exact were used to compare baseline information between groups.

The Overall PPOS score of the 18 items was computed as an unweighted mean. Two sub-scale scores “Caring” and “Sharing” were derived from the 18 items. Statistical descriptive for baseline (T1) PPOS scores (Overall, Sharing, Caring) was calculated stratified by participants’ characteristics – age, sex, ethnicity, profession and work experience. Ethnic group distribution in the study sample was too small for some groups for the differences to be meaningful. Hence separate t-test (one-way ANOVA) were performed only for sex, profession and work experience.

As the context and culture where the original cut-offs were developed were different (Krupat et al derived the cut-offs from a cohort of 177 doctors from New England who were predominantly White [26]), the cut-offs for this study were derived from the empirical data, hence contextualised to the population. The outcome variable (baseline PPOS scores) was ranked by mean scores and divided into thirds (high, medium, low) with a relatively similar number of subjects. This was the same method used in the original validation of the instrument where the mean scores were ranked and divided into three groups [26]. The cut-offs derived for the three categories based on our empirical data were: i) high: mean score of ≥ 4.50 (ii) medium: > 4.06 to < 4.50 (iii) low: mean of ≤ 4.06. An Ordinal Generalised Estimating Equation (GEE) model was used to analyse the ordinal PPOS variable over time as responses from participants were not independent over time, and the GEE model accounts for this correlation between responses. To examine the effects of the training on our outcomes, we ran a main effects model with interaction effects (Time X Sociodemographic (Sex, Profession, and Work Experience)). For each predictor (sex, profession, and work experience), all of its possible interactions were tested with a single test (Wald statistic). These interactions were included based on the literature and the professional clinical judgement of the study team.

## Results

### Characteristics of the Study Sample

A total of 482 participants underwent the training programme between August 2020 and November 2021, of which 460 (95%) participated in the study. For analysis purposes, we excluded non-healthcare professionals (n = 18). There was one duplicate entry and four missing responses, resulting in 437 participants who were health practitioners being included in the final analysis. Overall, 56 participants (12.8%) dropped out from the study (i.e. did not complete both surveys at T1 and T2). Due to the anonymous nature of the survey, reasons for drop-out were not captured as participants who dropped out could not be contacted. Characteristics of the study participants are detailed in Table 1.

**Table 1:** Characteristics of participants (N = 437) and baseline PPOS scores.


		MEAN (SD) SCORES		

CHARACTERISTICS	n (%)	OVERALL PPOS	SHARING SUB-SCALE	CARING SUB-SCALE

**All participants**	437 (100%)	4.28	4.13	4.43

**Age (mean, SD)**	**= 36.3 (9.60)**			

<30	112 (25.6%)	4.20 (.47)	4.01 (.57)	4.39 (.53)

30–39	217 (49.7%)	4.30 (.52)	4.16 (.64)	4.45 (.54)

40–49	57 (13.0%)	4.40 (.59)	4.27 (.79)	4.53 (.56)

>=50	51 (11.7%)	4.24 (.47)	4.13 (.54)	4.35 (.57)

		*p* = 0.09	*p* = 0.06	*p* = 0.31

**Sex**				

Male	97 (22.2%)	4.17 (.5)	3.94 (.6)	4.41 (.58)

Female	340 (77.8%)	4.31 (.52)	4.19 (.64)	4.44 (.54)

		*p* = 0.02*	*p* < 0.001*	*p* = 0.59

**Ethnicity**				

Chinese	324 (74.1%)	4.27 (.49)	4.10 (.62)	4.45 (.52)

Malay	47 (10.8%)	4.22 (.59)	4.13 (.71)	4.32 (.60)

Indian	48 (11.0%)	4.40 (.60)	4.33 (.69)	4.44 (.64)

Others	18 (4.1%)	4.32 (.49)	4.27 (.62)	4.38 (.50)

		*p* = 0.43	*p* = 0.10	*p* = 0.46

**Profession**				

Doctor	76 (17.4%)	4.33 (.44)	4.13 (.54)	4.54 (.48)

Nurse	148 (33.9%)	4.27 (.52)	4.19 (.64)	4.34 (.54)

Allied Health	213 (48.7%)	4.28 (.54)	4.09 (.67)	4.46 (.56)

		*p* = .64	*p* = .34	*p* = 0.02*

**Work experience (mean, SD)**	**= 11.84 (9.58)**			

<5	97 (22.2%)	4.22 (.50)	4.01 (.59)	4.43 (.52)

5–10	148 (33.9%)	4.24 (.49)	4.08 (.61)	4.39 (.54)

11–20	138 (31.6%)	4.41 (.56)	4.32 (.70)	4.51 (.54)

21–30	32 (7.3%)	4.23 (.48)	4.07 (.60)	4.38 (.58)

>30	22 (5.0%)	4.12 (.42)	3.95 (.45)	4.28 (.64)

		*p* = 0.007*	*p* = 0.001*	*p* = 0.24

**PPOS category**				

**High** (PPOS Score ≥ 4.50)	= 151 (34.6%)			

**Medium** (PPOS Score >4.06 to <4.50)	= 136 (31.1%)			

**Low** (PPOS Score ≤ 4.06)	= 150 (34.3%)			

**PCC endorsement**				

All five PCC statements	= 329 (75.3%)			


#PPOS, Patient-Practitioner Orientation Scale.

### Baseline PPOS Scores

Baseline PPOS scores (Table 1) collected before the training showed Caring scores were higher than Sharing scores. Females were more person-centred as demonstrated by higher Overall and Sharing Scores. Caring scores varied by profession. The caring scores according to the instrument used showed doctors had the highest scores, followed by allied health practitioners and then nurses. Overall and Sharing scores varied by work experience: those with 11 – 20 years of experience had higher person-centred scores than their newer or more experienced co-workers.

Based on our empirical data, the cut-offs derived for each category (high: ≥ 4.50, medium: > 4.06 to < 4.50; low: ≤ 4.06) are lower than those reported by Krupat et al [26] (high: ≥ 5.00, medium: > 4.57 to < 5.00; low: ≤ 4.57).

### PPOS Scores After Three Months Remained Significantly Higher than Baseline

**Figure 3:**
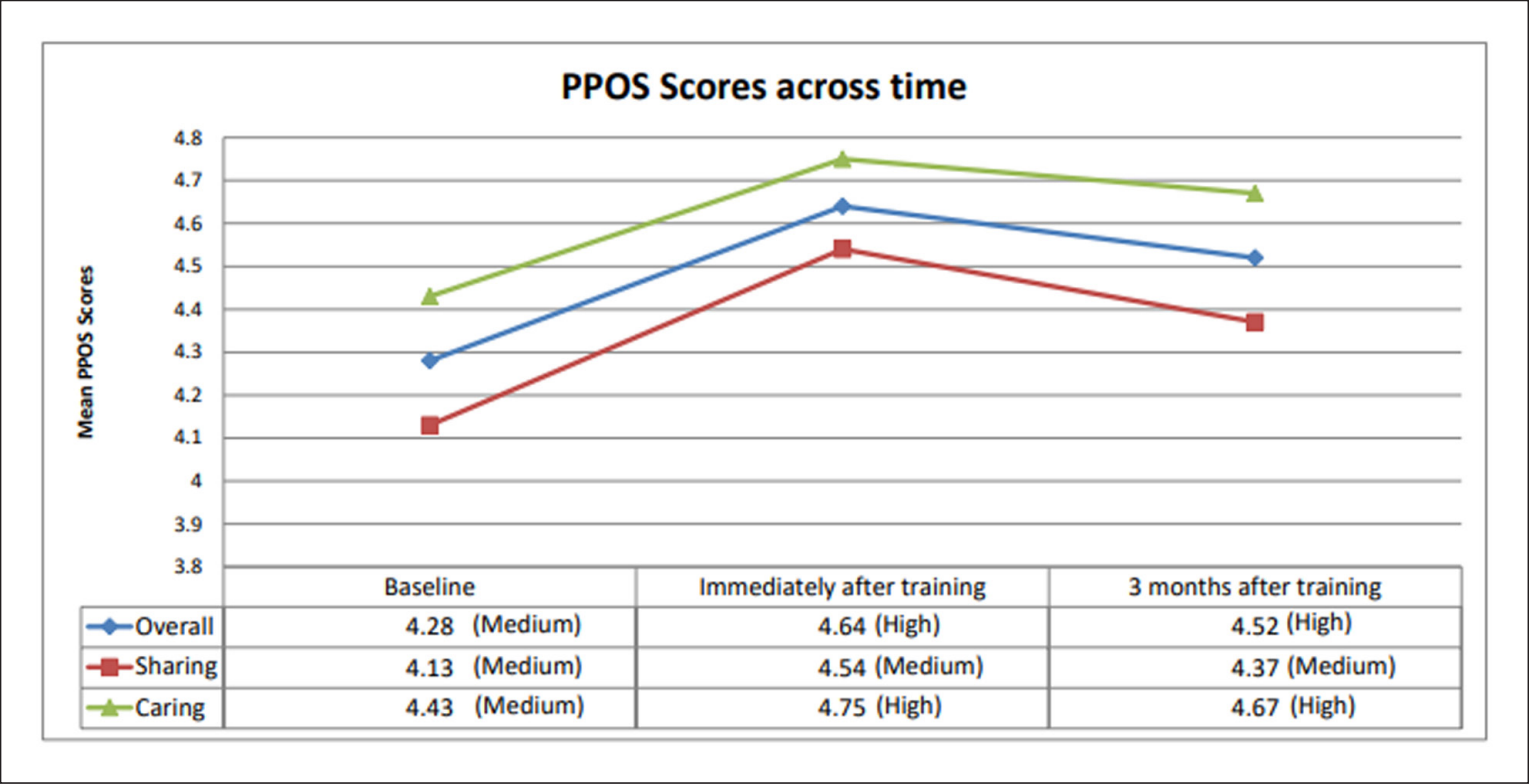
Relationship over time between PPOS scores (Overall, Sharing and Caring) and the advocacy training. #PPOS=Patient-Practitioner Orientation Scale.

Figure 3 shows the changes in PPOS scores for the Overall, Sharing and Caring sub-scales over time. Immediately after the advocacy training (T2), Overall, Sharing and Caring categorical scores increased from baseline (T1) (*p* < 0.001). Three months after the training (T3), these scores decreased from T2 (*p* < 0.001) but are still significantly higher than T1 (*p* < 0.001).

Participants who selected all five PCC statements rose from 75.3% to 92.7% (*p* < 0.001) immediately after the training.

#### Overall PPOS Scores

**Figure 4:**
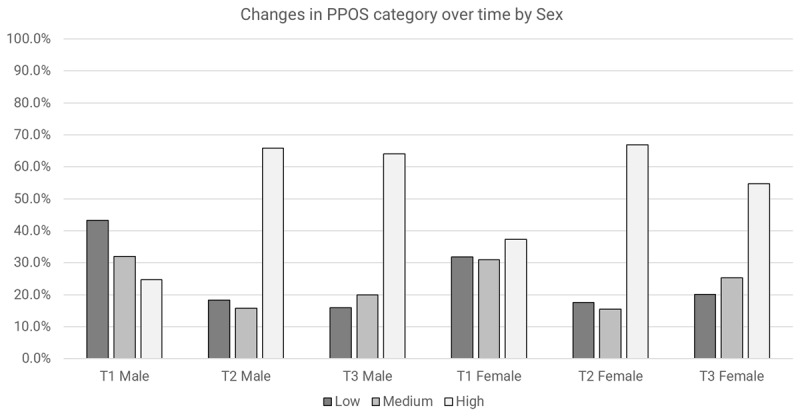
Changes in overall PPOS category over time by sex.

**Figure 5:**
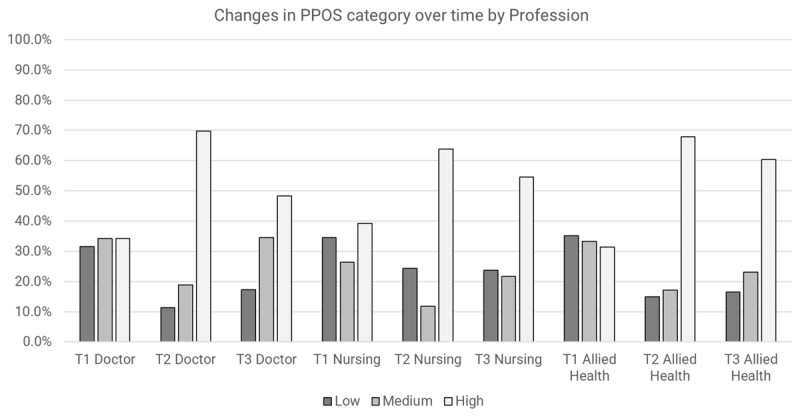
Changes in overall PPOS category over time by profession.

Both time and sex appeared to have an impact on Overall PPOS outcomes after adjusting for the other variables (main effects) (Table 2). There appeared to be increased odds of having a higher PPOS category over time: immediately after the training (T2), practitioners had 4.8 times the odds of having a higher PPOS category compared to baseline (OR = 4.8, 95% CI 2.36 to 9.92, p < 0.001). Females had 1.8 times the odds of having a higher Overall PPOS category compared to males (OR = 1.8, 95% CI 1.16 to 2.76, p = 0.008). Significant results for Wald’s test were observed for time X sex (p = 0.003), and time X profession (p = 0.033). Both males and females were more likely to have a higher Overall PPOS category over time (Figure 4). Individuals from all professions were more likely to have a higher Overall PPOS category over time, with a slightly greater increment of the effect at T3 for allied health professions (Figure 5).

**Table 2:** Main and interaction effects on PPOS outcomes (Overall, Sharing and Caring).


	OVERALL PPOS	95% CONFIDENCE INTERVAL			Sharing PPOS	95% CONFIDENCE INTERVAL			Caring PPOS	95% CONFIDENCE INTERVAL		
		
	OR	LOWER LIMIT	UPPER LIMIT	*P*-VALUE	OR	Lower limit	Upper limit	*P*-VALUE	OR	LOWER LIMIT	UPPER LIMIT	*P*-VALUE

**Sex**												

Male	1.00 (ref)				1.00 (ref)				1.00 (ref)			

Female	1.79	1.16	2.76	0.008*	2.13	1.34	3.38	0.001*	1.32	0.84	2.07	0.236

**Profession**												

Doctor	1.00 (ref)				1.00 (ref)				1.00 (ref)			

Nurse	0.68	0.39	1.19	0.177	1.00	0.58	1.75	0.990	0.48	0.28	0.85	0.012*

Allied Health	0.85	0.52	1.41	0.534	1.10	0.66	1.84	0.715	0.77	0.46	1.29	0.323

**Time**												

T1	1.00 (ref)				1.00 (ref)				1.00 (ref)			

T2	4.34	2.15	8.73	<0.001*	3.53	1.87	6.66	<0.001*	3.17	1.36	7.36	0.007*

T3	1.90	0.87	4.15	0.110	3.15	1.40	7.08	0.005*	2.72	0.89	8.28	0.078

Time*sex												

T2*Male	1.00 (ref)				1.00 (ref)				1.00 (ref)			

T2*Female	0.92	0.56	1.52	0.748	0.84	0.54	1.30	0.434	0.78	0.43	1.44	0.432

T3*Male	1.00 (ref)				1.00 (ref)				1.00 (ref)			

T3*Female	0.43	0.26	0.72	0.001*	0.54	0.31	0.93	0.026*	0.44	0.25	0.79	0.005*

Time*profession												

T2*Doctor	1.00 (ref)				1.00 (ref)				1.00 (ref)			

T2*Nurse	0.74	0.38	1.44	0.373	1.17	0.63	2.16	0.627	0.78	0.35	1.73	0.537

T2*Allied Health	0.88	0.46	1.68	0.706	1.31	0.74	2.33	0.358	0.92	0.41	2.05	0.837

T3*Doctor	1.00 (ref)				1.00 (ref)				1.00 (ref)			

T3*Nurse	1.72	0.91	3.24	0.093	1.22	0.60	2.49	0.576	0.87	0.36	2.12	0.762

T3*Allied Health	2.41	1.29	4.47	0.006*	1.30	0.67	2.53	0.432	1.04	0.41	2.62	0.933


#### Sharing Scores

After adjusting for the other variables, main effects were observed for time and sex. Immediately after the training (T2), care providers had **3.5 times** the odds of having a higher PPOS Sharing category compared to baseline (OR = 3.5, 95% CI 1.87 to 6.66, *p* < 0.001), and 3.2 times odds at 3-months (T3) compared to baseline (OR: 3.2, 95% CI 1.40 to 7.08, *p* = 0.005). Females appeared to have **2.1 times** the odds of having a higher PPOS Sharing category compared to males (OR: 2.1, 95% CI 1.34 to 3.38, *p* = 0.001). Tests of interactions were not significant, which means there is no interaction between the independent variables of time, sex, profession and work experience on Sharing scores.

#### Caring Scores

**Figure 6:**
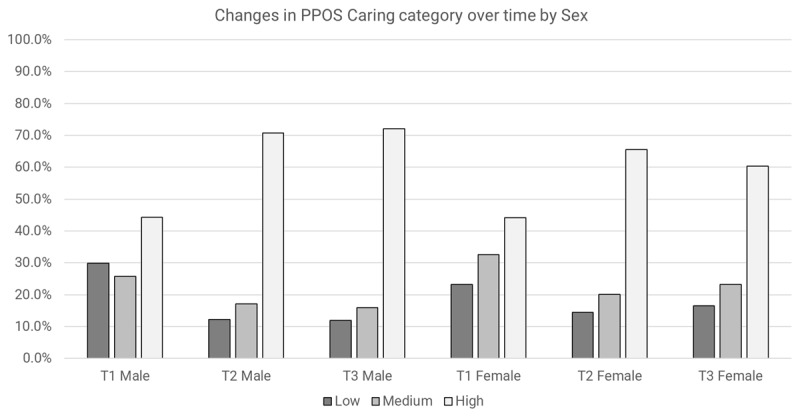
Changes in PPOS caring category over time by sex.

After adjusting for other variables, main effects were detected for time and profession. Immediately after the training (T2), care providers had **3.2 times** the odds of having a higher PPOS Caring category compared to baseline (OR = 3.2, 95% CI 1.36 to 7.36, *p* = 0.007). Compared to doctors, nurses had 0.48 times the odds of having a higher PPOS Caring category (OR = 0.48 95% CI 0.28 to 0.85, *p* = 0.012). Wald’s test was significant for time X sex (p = 0.021). Both males and females were more likely to have a higher PPOS Caring category over time (Figure 6).

## Discussion

In this study, we found that a single training session in PCC was associated with measurable improvements in person-centredness of health practitioners. After attending this advocacy training, participants were more likely to reach a higher Overall PPOS Category over time. The change in categories from T1 to T3 for Overall PPOS varied by sex and profession type, while the change in categories from T1 to T3 for PPOS Caring scores varied by sex. The proportion of health practitioners who accurately defined PCC increased immediately after the training. To our knowledge, this is one of the few studies of its kind to examine the relationship between PCC advocacy training and changes in person-centredness of health practitioners, particularly in the aspect of power sharing with service users (a key contributor to adherence to treatment) [7].

Building upon the conceptual model of a PCC innovation developed in an earlier study from our group [20 p^10^,^11^], we explicate the interaction between the intervention (in this case, the PCC advocacy training), context and role of social actors at the microsystem, mesosystem and macrosystem levels [39]. We relate the findings of this study to other published studies and discuss the implications for stakeholders in other contexts who similarly wish to promote a cultural shift towards PCC.

### Macrosystem: cultural and professional context – health practitioners were more predisposed to provide care than share power and control

We and other published studies across various contexts have found that the Caring aspect of PCC is more prevalent than the Sharing aspect of PCC. In our study, Caring Scores were higher than Sharing Scores across three time points. This may reflect a stronger pre-existing disposition towards the care aspect in medical consultations than to the aspects of equal sharing of power and control with service users, mirroring earlier studies elsewhere [21,22,26,27,32,37,40,41]. A study of person-centred attitudes among medical students in Singapore found similarly lower sharing scores [10]. The study postulated that factors such as traditions and cultures might be contributing reasons, where a more paternalistic approach still prevails [42]. The relationship between culture and workplace practice is a complex, multi-level relationship embedded in the specific context of a particular society [43] through institutional values, habits, traditions and processes [13]. Our empirical findings mirrored the findings in Lee et al’s study 14 years ago, where sharing scores are lower than caring scores [10]. The fact that little attitude shifts have been made over the years in the area of equal partnerships with service users may also reflect a dilemma attached to health practitioners, where a perceived sense of responsibility and control over patients’ outcomes may explain why “Sharing” does not dominate. This remains a concern in view that caring improves satisfaction but it is sharing that improves adherence [7], strengthens capacity for self-management [44,45] and enhances patient safety [46]. This finding is consistent with previous studies that found cultural change is hard to achieve and requires time, infrastructure, a systems approach and leadership [47].

### Mesosystem: Organisations as learning systems to sustain continuous improvements in practitioners

Our study revealed that training was associated with an increase in participants’ person-centred orientation, but this effect seems to wane somewhat with time and may benefit from training over time, as a “booster”, to reinforce person-centred beliefs and behaviours. Lacking a control group, we cannot attribute the shift in person-centred orientation to the training with certainty. However, a systematic review of studies on person-centredness training found positive effects on practitioner empathy and patients’ perception of practitioners’ attentiveness in clinical consultations after training [14]. The same review found that training of short duration (less than 10 hours) can be as successful as longer duration training [14]. Our findings indicate a possible attenuation of the training’s effect on Overall, Sharing and Caring Scores from T2 (immediately after training) to T3 (three months after training). This mirrors what previous studies found about the challenges of sustaining the outcomes [14,15]. Positioning the organisation as a learning health system [48] with shared vision and systems thinking may bring about collective team learning and build reinforcement and accountability among teams. At an organisational level, features of a learning system could be incorporated including (1) post-training engagements through a network to build trust and long-term relationships among practitioners that may reinforce a long-lasting effect [15] (2) policies and incentives to facilitate PCC at an organisational level. It is important to alternate between these learning sessions and workplace practice for feedback, reflection and assimilation, to reinforce the learning and cultural change [49].

### Microsystem: lower PCC literacy of health practitioners locally reflects the need for reflexivity to further augment training

The lower categorical cut-off scores (high, medium, low) derived from our empirical data compared to the scores generated by Krupat [26] implied a lower person-centred literacy locally compared to western counterparts who had similar challenges but evolved and advocated for coproduction as early as the 1970s [50]. Sweden demonstrated some success in focusing practitioners on person-centredness through the active involvement of service users, and teaching of a common language and system to all levels of staff [15]. These forms of coaching have contributed to improved team dynamics and morale at work [49,51]. Our empirical data showed encouraging results pointing in similar direction: even though Caring Scores were higher than Sharing Scores across the three time points (Figure 3), the degree of improvement was larger for Sharing than Caring immediately after training (odds of having a higher PPOS Category was 3.5 times for Sharing compared to 3.2 for Caring – Table 2). Addressing the issue of power sharing, Farr [52] identified possible approaches to connect with individual’s values and motivations which can result in long-lasting effects. Farr [52] suggested that reflexivity may be a mechanism for change in self and others.

### Microsystem: leverage on the evolving role of professionals to drive person-centred care

Our study found interaction effects between PPOS scores and profession type to be significant. Individuals from all professions were more likely to have a higher Overall PPOS category over time, with a slightly greater increment of the effect at T3 for Allied Health professions. This was an unexpected cross-interaction finding as most studies found little or no association between PPOS scores and the type of profession [27,37,53,54], including allied health professionals [22,55]. In the United States, allied health practitioners appear to have a narrow job scope with limited opportunities to expand their roles [56]. In recent times, there is an impetus to transform allied health practices to achieve better population health. Corroborating our findings and views, a study on leadership development for allied health found an increase in AHP’s abilities to lead and manage change to improve culture and safety through PCC practice [57]. The implications for this are that professional roles do evolve and change over time, which present opportunities for social actors to lead and drive person-centred movements.

### Microsystem: health practitioners’ perception of service users

Our findings that health practitioners are less predisposed to sharing power, control and information may reflect on how service users are perceived by care practitioners, prompting the need to better respond to inequalities and manage diversity to ensure the voices of marginalised and vulnerable service users are included [4]. For example, the study by Lee et al [10] postulated that lower sharing scores of medical students were associated with their perception that patients do not need the information. A key feature in the EN advocacy training, involvement of service users and its benefits in healthcare improvement efforts, is well documented in published research [58,59,60]. In our previous study, we found that service users provide a driving force that unites practitioners and enables organisations to adapt healthcare practices to the needs of services users [20], thereby increasing patient safety and care quality [61]. Drawing from published studies, it seems the application of PCC has yet to embrace strategic and mindful involvement of users. This is illustrated in the literature, for example by Farr [52] in her study of power dynamics in coproduction, who wondered if a diversity of groups is involved or the ‘more articulate and managerially experienced’? Structurally, are service users expected to fit into formal meetings within institutions and are they drawn into managerial agenda? In involving service users, it is important to be aware of categorical inequalities that exist due to the age, language, sex, education level etc. acting on individuals or groups, in any society. Through acknowledgement of the differences within service users, practitioners and between service users and practitioners, power imbalance can be better managed.

### Implications for further studies

In advocating for person-centred changes in attitudes and changed action in practice, this study has raised further questions about the interplay between and among practitioners and service users, and the interaction between the intervention, these social actors, culture, structure and the wider organisational context. We plan a follow-up qualitative study of participants’ experience and reflection on clinical application of the PCC philosophy, the observed impact on service users, and challenges faced. With this understanding, the authors will be better placed to further study the application of these attitudes in practice by measuring behaviours. The Four Habits Model is an example of measuring clinicians’ improvement in communication behaviour after training, in a consistent way [62,63]. Corroborating our study, research on the Four Habits Model also found system level influencers, such as support from senior leadership and involvement of service users, as critical [4,13].

### Strengths and Limitations

To our knowledge, this is one of the few studies of its kind to examine the relationship between a person-centred training – the PCC advocacy training – and healthcare practitioners’ person-centred attitudes and knowledge over time. It is a prospective study utilising convenience sampling based on the cohort who attended this training [64]. The resulting large sample size is a strength which allowed us to reveal and compare changes in PPOS scores in several subgroups. The measurement of results across three time points rather than merely after the training is a strength, and further studies could consider a longer timeline than three months after training.

Randomisation or the inclusion of a comparison group in the form of another group of health practitioners who did not go through the specific advocacy training would strengthen the internal validity in terms of our ability to attribute changes in person-centred attitudes to the training rather than to other causes such as a secular trend (towards person-centredness) or other changes in the work environment. Additionally, the study did not control for potential confounders in the study design or statistical analysis, which may limit the ability to draw causal inferences from the results. The study was conducted in a single setting and rather unique characteristics of a particular organisation in Singapore that may limit the generalisability of the findings to other healthcare settings or culture. However, by describing both the intervention (Figure 1) and context, the study promoted transferability through learning and adaptation where others design and try similar interventions that are adapted to their contexts.

The PPOS instrument though validated widely, has psychometric limitations such as treating data collected using the six-point Linkert scale as interval data which requires each score to be placed at an equal distance from one another. This was mitigated in our study by treating data as ordinal and deriving the cut-offs by ranking the study’s raw dataset. The limitation here is the lack of validation as our cut-offs were based only on the cohort of the current study.

The characteristics tested were not exhaustive and there is room to explore other possible independent variables not included in this study, such as socio-economic status, education, religious background which may contribute to stratification of the participant population to generate a better understanding of how the training “worked” and might be developed in the future [65,66].

## Conclusion

This empirical study reported on the attitudes of health practitioners towards PCC in a cultural context that privileges hierarchical relations. It found that PCC advocacy training was associated with higher PPOS scores among participating health practitioners, suggesting that health practitioners are responsive to PCC training. This finding corroborated integrated care implementation efforts to invest, involve and support the healthcare workforce. However, equalising power between providers and patients was harder to achieve through training alone, within a hierarchical structure and clinician-centric culture. The study also raised critical questions on how cultural awareness, attributes and role of practitioners, as well as organisational support structures including leadership support, at the macrosystem, mesosystem and microsystem levels, can further augment PCC advocacy training of health practitioners.

## Johan Thor

Published posthumously. Johan participated actively at all stages of the research and gave his consent for the revised version before he passed away from illness on 9 Oct 2023.
